# The Notch pathway regulates both the proliferation and differentiation of follicular cells in the panoistic ovary of *Blattella germanica*

**DOI:** 10.1098/rsob.150197

**Published:** 2016-01-13

**Authors:** Paula Irles, Nashwa Elshaer, Maria-Dolors Piulachs

**Affiliations:** 1 Institut de Biologia Evolutiva (CSIC-Universitat Pompeu Fabra), Passeig Marítim de la Barceloneta 37, 08003 Barcelona, Spain; 2 Facultad de Agronomía e Ingeniería Forestal, Pontificia Universidad Católica de Chile, Av. Vicuña Mackenna 4860, Macul, Santiago, Chile

**Keywords:** Notch, panoistic ovary, RNAi, oogenesis, cell proliferation, *Drosophila*

## Abstract

The Notch pathway is an essential regulator of cell proliferation and differentiation during development. Its involvement in insect oogenesis has been examined in insect species with meroistic ovaries, and it is known to play a fundamental role in cell fate decisions and the induction of the mitosis-to-endocycle switch in follicular cells (FCs). This work reports the functions of the main components of the Notch pathway (Notch and its ligands Delta and Serrate) during oogenesis in *Blattella germanica*, a phylogenetically basal species with panoistic ovary. As is revealed by RNAi-based analyses, Notch and Delta were found to contribute towards maintaining the FCs in an immature, non-apoptotic state. This ancestral function of Notch appears in opposition to the induction of transition from mitosis to endocycle that Notch exerts in *Drosophila melanogaster,* a change in the Notch function that might be in agreement with the evolution of the insect ovary types. Notch was also shown to play an active role in inducing ovarian follicle elongation via the regulation of the cytoskeleton. In addition, Delta and Notch interactions were seen to determine the differentiation of the posterior population of FCs. Serrate levels were found to be Notch-dependent and are involved in the control of the FC programme, although they would appear to play no crucial role in panoistic ovary oogenesis.

## 1. Introduction

Cell–cell communication is a basic activity of multicellular organisms, and regulates one of the most important events of development: cell differentiation. Certainly, Notch (N) signalling would appear to be one of the most important pathways involved in determining cell fate [1–3].

The core integral proteins of the Notch pathway in insects are the Delta (Dl) and Serrate (Ser) ligands of N (known as Delta-like and Jagged, respectively, in mammals), the N receptor (Notch 1–4 in mammals) and the transcription factor suppressor of hairless (Su-H) (CSL in mammals). Dl and Ser localize to the membranes of signal-sending cells, whereas N is located in the membranes of signal-receiving cells. Su-H joins to the N intercellular domain (NICD) in the nucleus, triggering the transcription of multiple target genes [4,5].

Notch has a pleiotropic function during the development of *Drosophila melanogaster* [4,6], acting as a general developmental factor in the direction of cell fate choices. The N gene was first described in *Drosophila* in 1919 by Mohr [7]; its mutation produces females with notched wings. Notch signalling has since been thoroughly studied in the development of the nervous system of *D. melanogaster* embryos. During lateral inhibition, high levels of Dl provide a neural precursor that sends a signal through the N receptor to neighbouring cells, inducing them to follow an epidermal fate [8]. The absence of Dl or N leads to neuroblastic hyperplasia [9]. Dl/N signalling also has an inductive role in eye and wing development [10], and participates in the determination of cell fate during myogenesis [11]. Additionally, N signalling is involved in the segmentation of the crustacean *Daphnia magna* [12].

The Notch pathway is also reported to be an important regulator of insect oogenesis in three holometabolan species with meroistic ovaries—*D. melanogaster* [13], *Apis mellifera* [14] and *Tribolium castaneum* [15]—and in the development of the panoistic ovary of the hemimetabolan insect *Blattella germanica* [16]. In the germarium of *D. melanogaster*, N signalling is associated with the establishment of the germ stem cell niche that regulates the number of cap cells, the niche size and the subsequent proliferation of the germ cells [17]. In the egg chamber of *D. melanogaster*, Dl passes the signal from the germline cyst to N, located in the somatic cells, for the differentiation of cell populations that finally affect the polarity of the oocyte [2,18,19]. Notch and Dl mutants show an excess of posterior FCs owing to a change in cell fate; this also affects the anterior–posterior polarity of the oocyte [13]. The breakdown in the differentiation of the subpopulations of FCs has a dramatic effect on the microtubule cytoskeleton, impairing the formation of the anterior–posterior axis [20]. Notch mutations also cause hyperplasia in the polar cell precursor at the expense of stalk cells during early oogenesis, leading to the fusion of the egg chambers [13]. Finally, the expression of a constitutively active N or Dl ligand in the *D. melanogaster* egg chamber, or overexpression of Dl in the germline, gives rise to long stalk-like structures that include the stalk itself as well as polar and undifferentiated FCs [2]. A similar interaction between Dl and N may occur in *A. mellifera* oogenesis, in which both ligand and receptor are localized to the oocytes and FCs [14].

The FCs must enter, and later exit, the proliferative stage in a very precise manner, steps in which N signalling actively participates. The absence of N activity in *D. melanogaster* FCs leads to prolonged mitosis at the expense of the endocycle [19,21,22], whereas in *T. castaneum* the absence of N activity triggers premature entrance into the endocycle [15]. The interplay between the Notch and Hippo pathways during the oogenesis of *D. melanogaster* and *B. germanica* [16,23–25] has also been studied. Unlike in *D. melanogaster*, in which the Hippo pathway promotes N signalling in the FCs [23], N expression is repressed by Hpo in *B. germanica*, triggering the mitosis-to-endocycle switch [16].

Information is still lacking, however, on the molecular mechanisms underlying the N signalling pathway, and how ligands and receptors interact during oogenesis in the ancestral panoistic ovary. Using the cockroach species *B. germanica* as a model, this work examines the role of N (BgN in this context) in the elongation of the ovarian follicle, and the part it plays in FC cycle status*.* Insights gained via RNAi-based analyses into the role of the canonical ligands Dl (BgDl) and Ser (BgSer) in the panoistic ovary of this cockroach are discussed.

## 2. Results

### 2.1. BgN regulates ovarian follicle elongation

**Figure 1.**
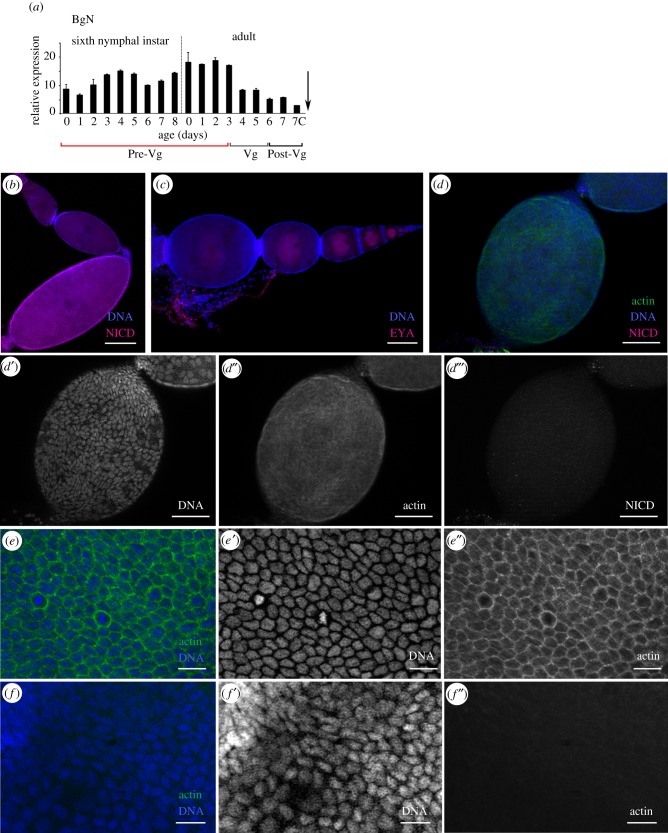
Expression pattern of BgN and cytoskeleton impairments in the ovaries of 0-day-old dsBgN-treated adult females. (*a*) BgN mRNA expression in ovaries of sixth-instar nymphs and adult females during the first gonadotrophic cycle, shows higher expression during the pre-vitellogenic period. The dashed line indicates the moult to adult, the arrow the oviposition time, and 7c the time of choriogenesis. The pre-vitellogenesis (Pre-Vg), vitellogenesis (Vg) and post-vitellogenesis (Post-Vg) stages are also indicated. Data represent copies of mRNA per 1000 copies of BgActin-5c (relative expression) and are expressed as means ± s.e.m. (*n* = 3). (*b*) Localization of BgN (NICD, magenta) in the cells placed in the germarium, in the stalk cells between the basal and sub-basal ovarian follicles, and in the FCs of the basal ovarian follicle of 0-day-old dsMock-treated adult females. (*c*) Ovariole from 0-day-old dsBgN-treated adult female shows the basal ovarian follicle with a spherical shape (the oocyte nucleus was labelled with the anti-Eya antibody). (*d*) Changes in FC planar polarity in basal ovarian follicles from 0-day-old dsBgN-treated adult females show (*d*′) nuclei, (*d*″) F-actin microfilaments and (*d*‴) NICD labelling (with no labelling). (*e*) Follicular epithelium in basal ovarian follicles from 0-day-old dsMock-treated females shows the FCs were mitotically active and the F-actin microfilaments were well distributed around the cell membranes: merged image of (*e*′) nuclei and (*e*″) F-actin microfilaments. (*f*) Follicular epithelium in basal ovarian follicles from 0-day-old dsBgN-treated females: merged image of (*f*′) nuclei and (*f*″) F-actin microfilaments. DAPI was used in DNA staining, and phalloidin-TRITC to stain the F-actin microfilaments. In all images, the posterior pole of the basal follicle is towards the bottom, except in (*c*) in which it is towards the left. Scale bars in (*e*,*f*): 20 µm; in (*b,d*): 50 µm and in (*c*): 100 µm.

BgN mRNA was strongly expressed in the ovaries during the pre-vitellogenic stage, from when the insects began the sixth (last) nymphal instar until becoming 3-day-old adults. At this latter time, which coincides with the beginning of vitellogenesis, BgN expression halved and remained close to this level until oviposition (figure 1*a*). Via the detection of the NICD, it has previously been shown that N is present at varying levels in the different ovarian follicles of *B. germanica* ovarioles [16]. In this work, BgN labelling appeared in the cells located in the germarium, in the stalk cells between the basal and sub-basal ovarian follicles, and in the FCs of the basal ovarian follicle (figure 1*b*). BgN expression was increased in mitotically active cells.

Because dsBgN treatment of newly emerged sixth-instar individuals of *B. germanica* causes high mortality during the imaginal moult [16], dsBgN was injected into 6-day-old nymphs just before the beginning of apolysis. The nymphs moulted correctly through to the adult stage, and the ovary was analysed in 0-day-old (freshly ecdysed) adults. The ovaries of these new adult females were affected to different degrees, and were classified as either having a strong, i.e. dsBgN-S (58%; figure 1*d*) or mild, i.e. dsBgN-M (42%; electronic supplementary material, figure S1) phenotype. dsBgN-S basal ovarian follicles were spherical in shape (figure 1*c*,*d*) rather than the ovoid forms usually observed in dsMock-treated females (figure 1*b*). The dsBgN-S basal ovarian follicles (0.30 ± 0.02 mm; *n* = 13) were significantly shorter (around 36%) than those of dsMock-treated ovarian follicles (0.47 ± 0.02 mm (*n* = 16); *p* < 0.0001; Mann–Whitney test), although their width was similar (0.23 ± 0.01 mm compared with 0.24 ± 0.02 mm; *p* = 0.06; Mann–Whitney test). The dsBgN-M basal ovarian follicles were slightly but significantly shorter than those of the dsMock-treated females (0.43 ± 0.04 mm (*n* = 15) compared with 0.47 ± 0.02 mm (*n* = 16); *p* = 0.0011; Mann–Whitney test), but again the width was similar (0.26 ± 0.02 mm compared with 0.24 ± 0.02 mm; *p* = 0.06; Mann–Whitney test; electronic supplementary material, figure S1). While the ovarian follicles of 0-day-old dsBgN-treated adult females showed a mixture of mild and strong phenotypes, 5 days later (i.e. as 5-day-old adults), all showed the strong phenotype. Hereafter, we refer only to these latter, strong phenotype ovarian follicles.

The architecture of the F-actin microfilaments in the ovarian follicles of 0-day-old dsBgN-treated adult females appeared disorganized, and they stained poorly with phalloidin-TRITC (figure 1*d*,*d*″). Alterations in the planar polarity of the FCs were apparent; most of the FCs grouped at the poles and were elongated with an anterior–posterior orientation, as revealed by their nuclear morphology (figure 1*d′*). No BgN labelling was detected in the ovarian follicles (figure 1*d*,*d‴*). At higher magnification, the changes in the FCs were more evident. The FCs in the basal ovarian follicles of dsMock-treated females were small, mitotically active (figure 1*e,e′*) and had no spaces between them, and the F-actin microfilaments were well distributed around the cell membranes (figure 1*e″*). However, in the dsBgN-treated females, FCs were scarce, their nuclei showed different sizes and morphologies compared with the controls, and some had a picnotic appearance (figure 1*f–f*″).

**Figure 2.**
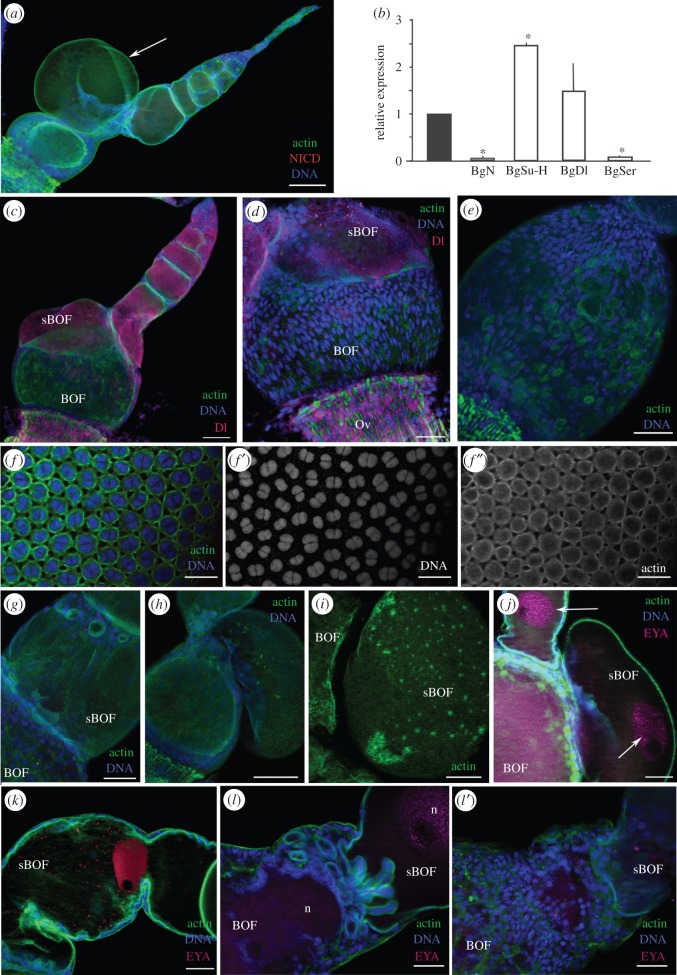
Depletion of BgN affects stalk formation in 5-day-old adult females. (*a*) Ovariole shows small ovarian follicles and a sub-basal oocyte that has broken the follicular epithelium, bulging out of the ovarian follicle. (*b*) Relative expression of BgN, BgSu-H, BgDl and BgSer mRNAs in ovaries from dsMock- and dsBgN-treated females, where the expression of BgN, BgSu-H and BgSer were significantly affected (asterisks). Data represent values normalized against the control (reference value = 1) (*n* = 3). (*c*) Ovariole from a dsBgN-treated adult shows the absence of the stalk. Labelling for BgDl (magenta) was localized to the cytoplasm of young oocytes; it was not detected in the basal ovarian follicle. (*d*) No labelling for BgDl was visible in the FCs of the basal ovarian follicle. Note that the cells in the oviduct are strongly labelled. (*e*) FC distribution in a basal ovarian follicle from a dsBgN-treated female. (*f*) Follicular epithelium from a basal ovarian follicle of a dsMock-treated female: merged image of (*f′*) shows the nuclei, and (*f″*) shows the F-actin microfilaments in the FCs. (*g*) Sub-basal ovarian follicle in a dsBgN-treated female shows the region of contact between adjacent follicles without the stalk. (*h*) Ovarian follicle from a dsBgN-treated female shows the extruded oocyte bulging into the region between two ovarian follicles. (*i*) Network of F-actin microfilaments in the membrane of the bulging oocyte. (*j*) The oocyte nucleus (labelled with anti-Eya antibodies, magenta) appeared in the bulge. (*k*) Sub-basal ovarian follicle from a dsBgN-treated female with a constriction in the central zone owing to F-actin concentration. The oocyte nucleus is strongly labelled for Eya (red). (*l,l′*) Region between the basal and sub-basal ovarian follicles without the stalk. (*l*) Oocyte membranes form multiple expansions in this area. (*l′*) Surface of (*l*) with the anomalous distribution of FCs. The oocyte nucleus (n) is labelled with anti-Eya (pink). sBOF, sub-basal ovarian follicle; BOF, basal ovarian follicle. In all images, the posterior pole of the basal follicle is towards the bottom left. Scale bars, 50 µm; except in *a,c* and *g*: 100 µm.

In 5-day-old dsBgN-treated adult females, the ovarian phenotype was more obviously affected (figure 2*a*). mRNA levels of BgN were depleted by some 98% (41.6-fold; P(H1) = 0.0001; REST analysis; figure 2*b*). Those of the N ligands were affected too: BgSer was downregulated by 11.6-fold (P(H1) = 0.0001; REST analysis), whereas BgDl was upregulated by 1.49-fold (P(H1) = 0.334) (figure 2*b*). BgSu-H, which is part of the sending signal complex that helps N reach the nucleus, was also upregulated 2.5-fold (P(H1) = 0.0001; REST analysis; figure 2*b*). The depletion of BgN resulted in basal ovarian follicles 82% shorter than those of the controls (*p* < 0.0001; figure 2*a*,*c*; see also [16]), and which maintained the spherical shape observed in 0-day-old dsBgN-treated females (figure 1*f*). A typical feature seen in the insects of this age was the reduction (even the absence) of the stalk between the ovarian follicles (figure 2*a*,*c,d*), a consequence of the absence of differentiated stalk cells in both the anterior region of the basal ovarian follicle and the posterior region of the sub-basal ovarian follicles (figure 2*c,g*).

The depletion of BgN mRNA in 5-day-old dsBgN-treated adult females resulted in the absence of BgN protein labelling in all ovarian follicles (figure 2*a*). BgDl was present in the younger oocytes (from the sub-basal follicles to the germarium), and extended throughout the cytoplasm (figure 2*c*), but no labelling for BgDl was seen in the basal ovarian follicles (figure 2*c*,*d*). The FCs at the poles were still mononucleated, and most of the nuclei maintained an elongated shape giving the cells an anterior–posterior orientation (figure 2*d,e*). The FCs located in the central region of the ovarian follicle, however, showed nuclei more rounded in shape. Finally, the majority of FCs in all locations showed a nucleus with a picnotic appearance (figure 2*d*). Conversely, in 5-day-old dsMock-treated adult females, the FCs were binucleated and had entered the endocycle (figure 2*f,f*′), and the F-actin microfilaments associated with the cell membranes had lateral extensions connecting the cells (figure 2*f*″), which showed patency. In dsBgN-treated adult females, the F-actin microfilaments (figure 2*c–e*) were not associated with FC membranes, and were distributed like a thin mesh covering the oocyte surface. They appeared more concentrated, however, in certain areas of the follicular epithelium.

The absence of stalk cells, the changes in FC morphology observed and the modifications of F-actin distribution precluded the normal growth of ovarian follicles. The sub-basal ovarian follicles of the 5-day-old dsBgN-treated adult females commonly showed overgrowth that broke the follicular epithelium to produce an external bulge hanging from the ovariole (figure 2*a*,*h–j*). In some cases, this bulge contained the oocyte nucleus (figure 2*j*). No FCs were seen covering these structures, and F-actin microfilaments associated with the oocyte membrane were detected as spots spreading over its surface (figure 2*i*). Occasionally, the anomalous distribution of the F-actin microfilaments constrained the elongation of the sub-basal ovarian follicles (figure 2*k*). In the regions connecting two ovarian follicles, cell membranes emerging from the basal follicle appeared, extending and invading the sub-basal follicles (figure 2*l*).

These results show that BgN is important for oocyte growth and maturation. BgN participates in ovarian follicle elongation, acting on the cytoskeleton network and regulating the anterior–posterior axis.

### 2.2. BgN maintains the follicular cells in a non-apoptotic stage

**Figure 3.**
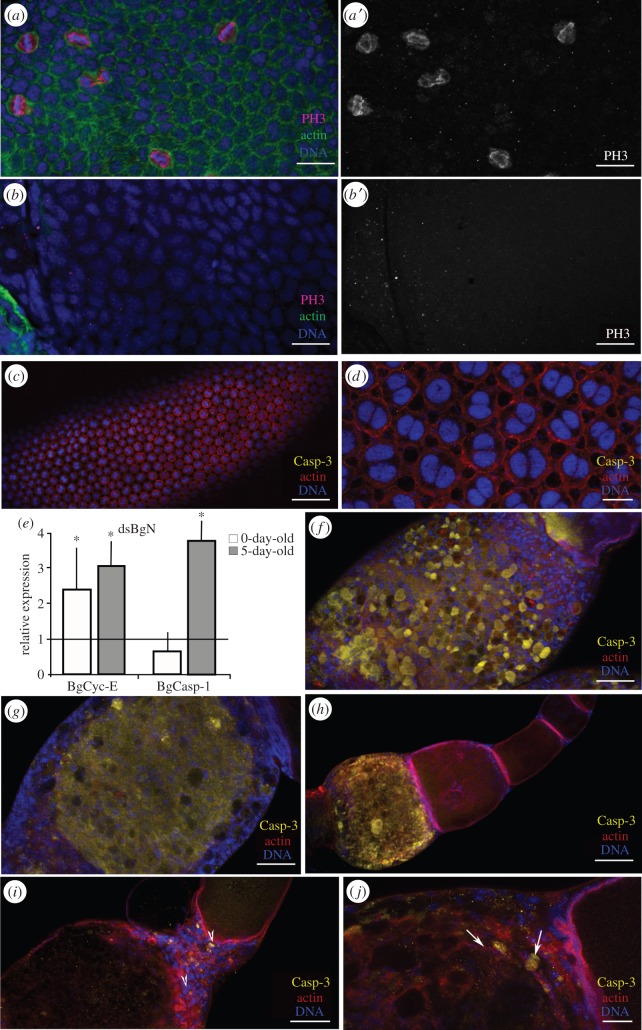
Mitosis stops and apoptosis is triggered earlier in FCs of dsBgN-treated females. (*a–b*) Follicular epithelium from 0-day-old dsBgN-treated adult females shows mitotic cells labelled with anti-PH3 (magenta). (*a,a*′) dsMock-treated and (*b,b*′) dsBgN-treated females show no detectable labelling. (*c–g*) Ovarian follicles from 5-day-old dsBgN-treated adult females. (*c*) Basal ovarian follicle from dsMock-treated female. (*d*) Close-up of the follicular epithelium labelled for caspase-3 (yellow). (*e*) Relative expression of BgCyc-E and BgCasp-1 in 0-day-old and 5-day-old dsBgN-treated females. The asterisks denote significant difference. Data represent values normalized against the control (reference value = 1) (*n* = 3). In 5-day-old dsBgN-treated adult females the morphology of the ovarian follicles suggested that they had become prematurely apoptotic. (*f*) Follicular epithelium in a basal ovarian follicle from a 5-day-old dsBgN-treated female, and (*g*) optical section of the same follicle: the ooplasm labelled for caspase-3 can be clearly seen. (*h*) Ovariole from a 5-day-old dsBgN-treated female: the basal ovarian follicle is strongly labelled for caspase-3. (*i,j*) Stalk region between basal and sub-basal ovarian follicles from a dsBgN-treated female: labelling for cleaved caspase-3 was concentrated in zone close to the stalk (*i*), and the labelled cells localized to the anterior part of the basal ovarian follicle (*j*; arrows). F-actin microfilaments (green in *a–b′* and red in *c–j*) stained with phalloidin-TRITC. Nuclei (DNA) stained with DAPI (blue). Scale bars, panels *a,b,d,j*: 20 µm; *c,h:* 100 µm; *f,g,i*: 50 µm.

In the basal ovarian follicles of the dsMock-treated adult females, the FCs were mitotically active during pre-vitellogenesis (figure 3*a,a′*). Cytokinesis was halted, and the endocycle begun when vitellogenesis started (occurring in 3-day-old adult females). From this moment, the FCs then became binucleated and polyploid (figures 2*f* and 3*d*). As seen in the 0-day-old dsBgN-treated adult females, the depletion of BgN resulted in the absence of mitosis in the FCs, as revealed by PH3 labelling (figure 3*b,b*′). Cyclin-E (BgCyc-E) levels were therefore checked by PCR to determine whether the FCs were entering the endocycle. mRNA levels of BgCyc-E were measured in 0-day-old (mitosis stage) and 5-day-old (endocycle stage) dsMock-treated and dsBgN-treated adult females, and 2.4-fold (P(H1) = 0.0001; REST analysis) and 2.6-fold (P(H1) = 0.036; REST analysis) upregulations recorded for these different dsBgN-treated females, respectively (figure 3*e*). Moreover, the morphology of the ovarian follicles in both the 0- and 5-day-old dsBgN-treated adult females suggested that they had become prematurely apoptotic. The expression of the effector caspase-1 (BgCasp-1) was therefore measured (figure 3*e*). In the ovaries of 0-day-old dsBgN-treated adult females it was found not to be affected, but in those of 5-day-old females its expression increased 3.2-fold (P(H1) = 0.013; REST analysis). Caspase activity in the ovaries was localized using an anti-cleavage caspase-3 antibody. No labelling was detected in the ovarian follicles of dsMock-treated females (figure 3*c,d*). However, in 5-day-old dsBgN-treated females, half of the ovarioles (54.5%; *n* = 22) showed extremely strong caspase-3 labelling in the basal ovarian follicles, the FCs and the ooplasm (figure 3*f,g*). It is worth noting that, among the ovarian follicles, only the basal type were labelled (figure 3*h*); no labelling of any younger follicles was seen. In the remaining dsBgN-treated ovarian follicles (45.5%; *n* = 22), very faint caspase-3 staining (figure 3*i*,*j*) was detected in groups of FCs mainly in the anterior region of the follicle and the transition zone between the basal and sub-basal follicles. Moreover, in agreement with the negative results for BgCasp-1 expression in 0-day-old dsBgN-treated females, no caspase-3 labelling was detected at this age (not shown).

Taken together, these results show that BgN regulates the cell cycle, maintaining, in the basal ovarian follicle, the FCs in a proliferative and non-apoptotic stage.

### 2.3. Expression of Notch ligands in the ovary of *Blattella germanica*

**Figure 4.**
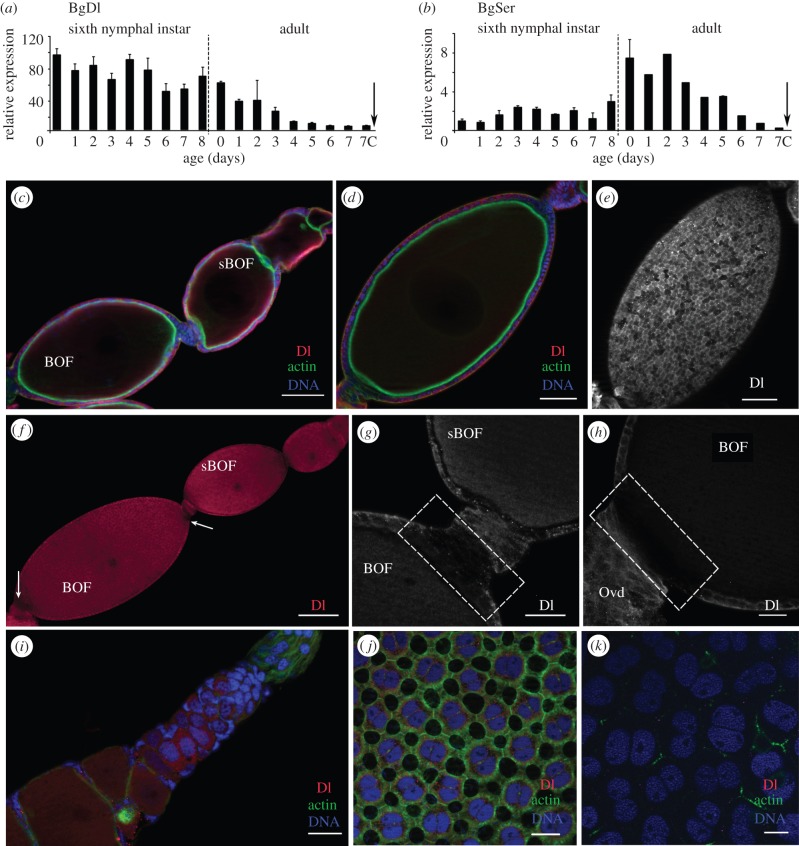
BgDl and BgSer in the ovary of *B. germanica*. (*a*,*b*) mRNA expression of BgDl (*a*) and BgSer (*b*) in ovaries from sixth-instar nymphs and adult females during the first gonadotrophic cycle, shows expression patterns with complementary profiles along nymphal stage. The dashed line indicates the moult to adult, the arrow the oviposition time, and 7c the time of choriogenesis. Data represent copies of mRNA per 1000 copies of BgActin-5c (relative expression), and are expressed as means ± s.e.m. (*n* = 3). (*c–k*) Immunolocalization of BgDl (magenta) in the ovaries. (*c*) Section (optical) of a 0-day-old sixth-instar nymph ovariole shows Dl labelling in the FCs of all the ovarian follicles. (*d*) Optical section and (*e*) FCs of a 5-day-old sixth-instar nymph basal ovarian follicle show Dl labelling in a more basal location in the FCs of all the ovarian follicles. (*f*) Ovariole from a 0-day-old adult shows the absence of BgDl labelling in the posterior FCs of all follicles (arrows). Detail of the anterior (*g*) and posterior poles (*h*) in a basal ovarian follicle from a 0-day-old dsMock-treated adult, shows the absence of labelling for BgDl in the FCs (dashed box). (*i*) Germarium from a 0-day-old dsMock-treated adult female shows the cells labelled for BgDl. FCs from (*j*) 5-day-old and (*k*) 7-day-old dsMock-treated adult females show a decrease of Dl labelling as the female is older, becoming even undetectable just before the oviposition. F-actin (green) was stained with phalloidin-TRITC, DNA with DAPI (blue). BOF, basal ovarian follicle, sBOF, sub-basal ovarian follicle. In all images, the posterior pole of the ovarian follicle is towards the bottom left. Scale bars, (*c*–*e*): 50 µm; (*f*): 100 µm; (*g*–*k*): 20 µm.

BgDl and BgSer, the canonical ligands of N, showed expression patterns with complementary profiles during the sixth nymphal instar, suggesting changing ligand availability for the BgN receptor. The expression of BgDl in the ovaries of sixth-instar nymphs showed slight variations over this developmental stage (figure 4*a*). However, the expression of BgDl during the adult stage fell continuously until the time of oviposition.

BgSer was expressed in the ovary at lower levels than BgDl, in both the sixth nymphal instar and in the adult stage. However, expression increased after the imaginal moult, coinciding with the mitotic programme in the FCs. After this moment, and matching with the switch of the FCs to the endocycle, the expression of BgSer declined gradually until the time of oviposition (figure 4*b*).

BgDl was immunolocalized in the ovaries using an antibody against the Dl extracellular domain of *D. melanogaster*. In the ovarioles of 0-day-old sixth-instar nymphs, BgDl appeared in the FCs of all the ovarian follicles (figure 4*c*), in general occupying a basolateral position. In the oocyte, however, it was detected close to the membrane. Later, in 5-day-old sixth-instar nymphs (figure 4*d,e*), it was observed in a more basal location in the FCs of all the ovarian follicles. A patchy distribution was seen for the entire follicular epithelium, with many cells showing no BgDl labelling at all (figure 4*e*). Remarkably, in both 0- and 5-day-old sixth-instar nymphs, no BgDl labelling was seen in the FCs at either the posterior or anterior pole in any developing ovarian follicle throughout the vitellarium (figure 4*c,d*).

In 0-day-old dsMock-treated adult females, the absence of BgDl labelling at the poles of the basal ovarian follicles was apparent (figure 4*f,* arrows). At the anterior pole (figure 4*g*), no BgDl was detected in the most anterior FCs, or in the stalk cells in contact with them. Similar observations were made at the posterior pole in those cells close to the pedicel (figure 4*h*). However, in the younger oocytes in the vitellarium and germarium, and indeed in some germ stem cells, BgDl appeared to be distributed throughout the cytoplasm (figure 4*i*). In 5-day-old dsMock-treated adult females, labelling in the FCs of the basal ovarian follicle was weak, but spread over the cytoplasm and nuclear membrane (figure 4*j*). During post-vitellogenesis, in 7-day-old dsMock-treated adult females, the decline in BgDl was more obvious, until becoming practically undetectable just before oviposition (figure 4*k*).

### 2.4. Depletion of BgDl and BgSer prevents the proper maturation of the ovarian follicle

To determine whether the relationship between the receptor (BgN) and their canonical ligands (BgDl and BgSer) is conserved in the panoistic ovaries of *B. germanica*, BgDl and BgSer were depleted by injecting the corresponding dsRNAs into 6-day-old sixth-instar nymphs and observing the phenotype in the adult stage.

**Figure 5.**
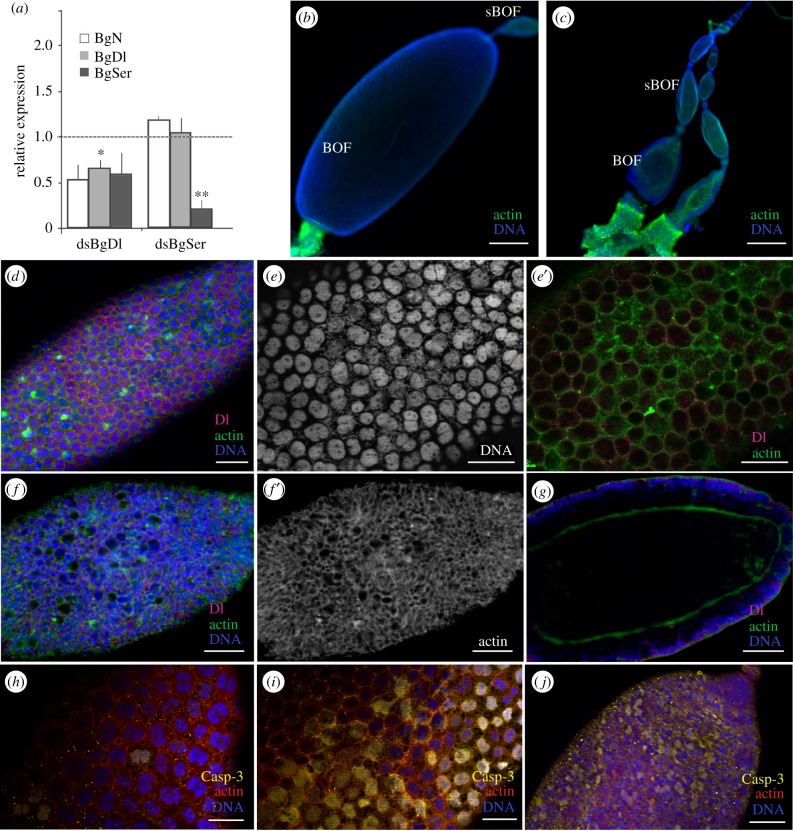
BgDl and BgSer affect the development of the basal ovarian follicle, inducing cell death in 5-day-old adult females. (*a*) mRNA expression levels of BgN, BgDl and BgSer in ovaries from dsBgDl- and dsBgSer-treated females. Data represent values normalized against the control (reference value = 1) (*n* = 3). The asterisks mean significant differences. (*b–j*) Ovarian follicles from 5-day-old dsBgDl-treated adults. (*b*) Follicle shows partial growth and (*c*) smaller ovarian follicles. (*d*) Follicular epithelium from a basal ovarian follicle shows partial growth, with some FCs showing Dl labelling. Detail of the follicular epithelia shows the nuclei from FCs labelled with DAPI (*e*), and the F-actin microfilaments (*e*′) that appeared to accumulate in the cytoplasm in those FCs lacking Dl labelling. (*f–f*′) Small basal ovarian follicle shows BgDl labelling and the F-actin microfilaments (*f*′) in the FCs. (*g*) Optical section of (*f*) shows the oocyte membrane detached from the follicular epithelia. (*h*–*j*) Immunolocalization of caspase-3 (yellow) in the basal ovarian follicle from dsMock- (*h*) and dsBgDl-treated females (bigger (*i*) and small (*j*) ovarioles, with a similar number of labelled cells). The F-actin microfilaments were stained with phalloidin-TRITC (green: *b*–*g* and red: *h*–*j*). DNA was stained with DAPI (blue). BOF, basal ovarian follicle and sBOF, sub-basal ovarian follicle. The posterior pole of the basal follicle is towards the bottom left. Scale bars, 50 µm, except in (*b* and *c*) 200 µm and (*e–e*′) 25 µm.

In the ovaries of 5-day-old dsBgDl-treated adult females, BgDl mRNA levels were significantly reduced (1.5-fold; P(H1) = 0.0001; REST analysis), whereas the expression of BgN and BgSer showed a trend towards decreasing (figure 5*a*). Conversely, in dsBgSer-treated adult females of the same age, the levels of BgSer mRNA in the ovaries were strongly reduced (4.5-fold; P(H1) = 0.0001; REST analysis; figure 5*a*) but the expressions of BgDl and BgN were unaffected. Further, all other target genes of the Notch pathway were affected by the depletion of the ligands (electronic supplementary material, figure S2). In dsBgDl-treated females, again 5-day-old, the size of the basal ovarian follicles showed notable intra-individual variation. Compared with dsMock-treated females (1.55 ± 0.08 mm; *n* = 12), some ovarian follicles partially grew (1.14 ± 0.19 mm (*n* = 7); *p* = 0.0006; Mann–Whitney test; figure 5*b*), and some were smaller (0.45 ± 0.06 mm (*n* = 14); *p* < 0.0001, Mann–Whitney test; figure 5*c*). These smaller ovarian follicles showed a fragile appearance, were closely connected and appeared tangled with the *tunica propria*.

In the basal ovarian follicles that grew partially, BgDl was detected in some FCs (figure 5*d*). In those FCs lacking BgDl labelling, the distribution of F-actin microfilaments appeared modified; they accumulated in the cytoplasm (figure 5*e*′,*i*″). In general, these basal ovarian follicles did not show patency (figure 5*e,e*′) and not all the FCs were binucleated (figure 5*e*); this contrasts with that seen in dsMock-treated females, in which all the FCs were binucleated (figure 4*j*). Moreover, the nuclei showed condensed chromatin, suggesting that these cells might be dying prematurely, as also indicated by caspase-3 labelling (figure 5*i*). Further, in the smaller basal ovarian follicles of 5-day-old dsBgDl-treated females, the FCs were smaller, had an irregular shape, and were mononucleated (figure 5*f*). The F-actin microfilaments appeared randomly distributed (figure 5*f′*), affecting the planar polarity of the epithelium and leaving large spaces between the cells. In addition, the oocyte membrane was generally detached from the FCs (figure 5*g*). Despite the severe defects of this phenotype, low levels of BgDl were detectable in some FCs (figure 5*g*). Although the morphology of these ovarian follicles suggests they were dying (figure 5*g*), the number of caspase-3 labelled FCs was more or less the same as that seen in the oocytes that partially grew (figure 5*i,j*).

In 5-day-old dsBgSer-treated adult females, the mRNA levels of BgSer were dramatically depleted. However, the basal follicles reached a similar length (1.6 ± 0.15 mm (*n* = 13); *p* = 0.42; Mann–Whitney test) to those of dsMock-treated females, and the morphological phenotype was the same as that of these controls. However, the FCs were significantly shorter (20.8 ± 0.37 µm (*n* = 27); *p* < 0.001; Mann–Whitney test) than in the dsMock-treated females (25.4 ± 0.6 µm (*n* = 37); electronic supplementary material, figure S3), and some FCs in the basal ovarian follicles of the dsBgSer-treated females showed clear caspase-3 labelling, indicating them to be apoptotic (electronic supplementary material, figure S4).

### 2.5. Unlike that seen with BgN depletion, RNAi of BgDl and BgSer revealed the stalk structure in the basal ovarian follicles

**Figure 6.**
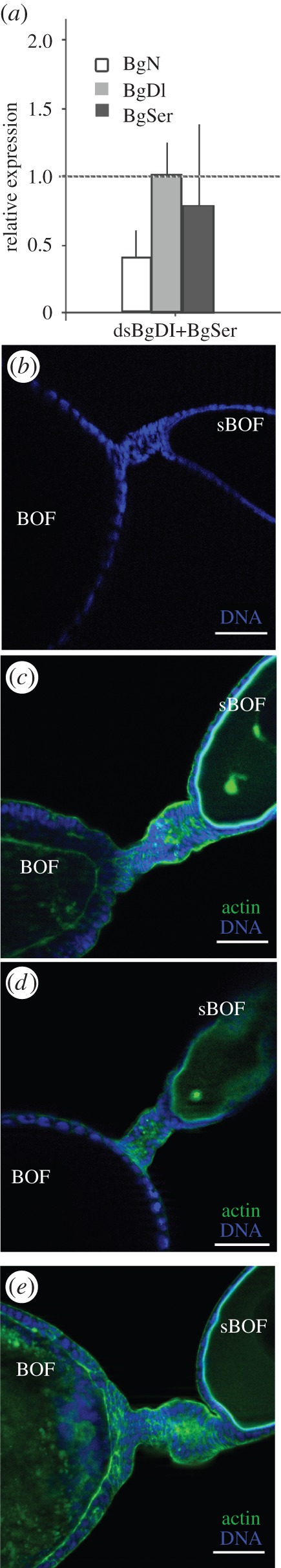
Depletion of BgDl and BgSer did not impede stalk formation. (*a*) mRNA expression levels of BgN, BgDl and BgSer in ovaries from 5-day-old dsBgDl + dsBgSer-treated females. Data represent values normalized against the control (reference value = 1) (*n* = 3). (*b–e*) Detail of the stalk structure connecting the basal (BOF) and the sub-basal (sBOF) ovarian follicles in 5-day-old dsMock-treated (*b*), dsBgDl-treated (*c*), dsBgSer-treated (*d*) and dsBgDl + dsBgSer-treated (*e*) females. The stalk was always formed in treated females and sometimes it is even longer than in dsMock-treated females. The F-actin microfilaments were stained with phalloidin-TRITC (green), and DNA with DAPI (blue). The posterior pole of the basal follicle is oriented towards the bottom left. Scale bars, 50 µm.

It was expected that depleting the ligands of BgN would also lead to the absence of the stalk that resulted from BgN depletion (figures 1 and 2). However, and although the mRNA expression of BgDl and BgSer was depleted, the stalk formed in a manner similar to that seen for the dsMock-treated females; indeed, sometimes it was even longer (figure 6*b–d*). With the aim of enhancing the expected interference, both dsBgDl and dsBgSer were injected at the same time in 0-day-old adult females, and follicle development observed 5 days later. In the ovaries of these double-treated females, the expression of BgN showed a trend to reduce by about half, BgDl levels were not affected and BgSer levels showed notable variation (figure 6*a* and electronic supplementary material, figure S4). In half of the treated specimens BgSer expression was significantly downregulated (P(H1) = 0.0001; REST analysis), whereas in the other half it was increased (P(H1) = 0.0001; REST analysis, electronic supplementary material, figure S5). However, the same ratio of strong-to-mild phenotypes was observed as for dsBgDl treatment alone (not shown), and the stalk was similar—indeed, it was sometimes even longer than those seen in controls (figure 6*e*).

These results show that reducing BgDl mRNA levels by half is not enough to endanger the formation of the stalk structure, and that the BgSer ligand is not necessary for its production.

### 2.6. The Notch pathway is involved in the differentiation of follicular cell populations at the poles

**Figure 7.**
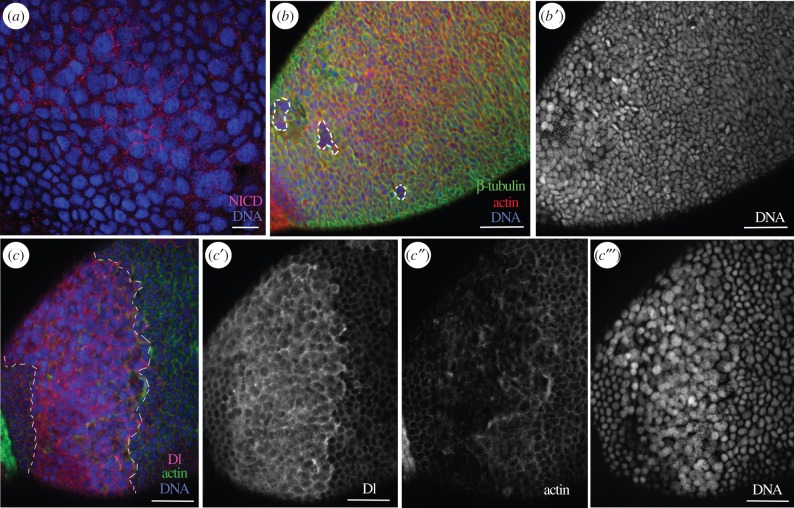
The BgN receptor and BgDl ligand determine the differentiation of posterior FCs. In 5-day-old dsBgHpo-treated adult females, the FCs in the posterior pole showed changes in their morphology and overexpressed NICD (*a*). Some FCs in this area lacked β-tubulin and F-actin (*b,* dashed frames). (*b*′) The nuclei of the latter cells were round in shape and bigger. (*c–c*‴) BgDl overexpression in the band of FCs (between the dashed lines), sourrounding the posterior FCs (*c*); (*c*′) Dl labelling was detected in the posterior FCs where usually it is not present; (*c*″) actin staining; shows the microfilaments clearly affected in the posterior region and (*c*‴) the nuclei of these FCs were bigger and with a differential staining intensity for DAPI compared to the rest of FCs. The F-actin microfilaments were stained with phalloidin-TRITC (red in *b* and green in *c*), DNA with DAPI (blue). The posterior pole of the basal follicle is orientated towards the bottom left. Scale bars, 20 µm in (*a*) and 50 µm in (*b*,*c*).

The absence of BgDl protein in the FCs at the basal ovarian follicle poles (figure 4), together with the changes in the distribution of these FCs observed after BgN depletion (figure 1), suggests that the Notch pathway is involved in the differentiation of specific FC populations. Moreover, BgN appears to be still active in FCs after BgHpo depletion, as was assessed by the strong NICD labelling in the posterior FCs (figure 7*a*; see also [16]). Further, these FCs showed differences in shape and pattern of distribution compared with dsMock-treated controls (figure 7*b,c*, dashed area). In some groups of FCs, the cytoskeletal proteins β-tubulin (figure 7*b*) and F-actin (figure 7*c*″) were almost absent. The nuclei of these cells were also larger and they showed different DNA staining compared with controls (figure 7*b*′,*c*‴). In the band of cells surrounding the posterior FCs, BgDl protein was highly overexpressed (figure 7*c*,*c*′). Remarkably, BgDl was detected in the posterior FCs; this ligand was not usually detected in these cells.

These results show that BgDl interacts with BgN in the differentiation of a specific type of posterior FCs.

## 3. Discussion

In the meroistic polytrophic ovary of *D. melanogaster*, the activation of N via Dl signalling triggers the switch from mitosis to the endocycle [19,21]. However, an ancestral role for the Notch pathway has recently been revealed in the meroistic telotrophic ovary of *T. castaneum* [15] and in the panoistic ovary of *B. germanica* [16]. In these less modified ovarian models, low level N signalling in the FCs is crucial in triggering the transition from mitosis to endocycle. In *B. germanica,* N activity is under the control of Hpo [16], as the depletion of BgHpo results in the maintenance of N activity, which keeps the mitotic programme of FCs along the first gonadotrophic cycle. This prolonged mitotic state was counteracted when N was depleted in these females with low levels of BgHpo, thus confirming the ancestral role of N in FC mitotic–endocycle switch [16].

The expression of N during oogenesis in *B. germanica* [16] is similar to that seen in *A. mellifera* [14] and *T. castaneum* [15], occurring both in germ and in somatic cells. BgN labelling disappears when the follicles have left the germarium, only to appear once more in oocytes that are ready to mature [16]. This is similar to that described for the *A. mellifera* ovary [14], but different to that seen in *D. melanogaste*r, in which FC expression of N is maintained until oogenesis is complete [26].

As in the meroistic telotrophic ovary of *T. castaneum* [15], the present results show N signalling to be needed in the panoistic ovary of *B. germanica* in order to maintain FCs mitotically active, and to keep them in an immature-like state. The entrance of the FCs into the endocycle usually requires the arrest of factors promoting mitosis, such as Cyclin-B, plus the phosphorylation of histone-3 and the upregulation of endocycling components such as Cyclin-E and/or Cyclin-D [27]. However, the depletion of BgN causes mitosis in the FCs to cease, preventing entry into the endocycle, as seen by the small size of the cells and their very small nuclei. Further, although Cyclin-E is overexpressed, strong caspase-1 expression suggests that these cells prematurely began programmed cell death; this was further confirmed by the presence of caspase-3 in the basal ovarian follicles of 5-day-old dsBgN-treated adult females. Remarkably, the apoptotic marker is only detected in the basal follicle and in stalk cells, where BgN is usually expressed. Whether this occurs because they fail to enter the endocycle, or because the FCs remain sensitive to other developmental signals when BgN is depleted, remains to be clarified. However, it has been described that the Notch pathway modulates the response to apoptosis in many developing organisms, connecting through crosstalk with other signalling pathways [28]. Moreover, in *D. melanogaster* oogenesis, N has an indirect role repressing apoptosis in the FCs after stage 6 [29]. Thus, decreasing BgN levels are required for FCs to transition to the endocycle, whereas N signalling is necessary for the survival of the maturing ovarian follicle.

The disorganization of the F-actin microfilaments after almost complete BgN depletion hinders the proper elongation of the follicle, resulting in the formation of spherical ovarian follicles. A similar phenotype has been observed in the egg chambers of *D. melanogaster* mutants for Fat2 cadherin; these mutants produce eggs spherical in shape and which have defects in the planar polar organization of the F-actin microfilaments, preventing egg elongation [30]. In addition, the interplay between the Notch and EGFR pathways maintains the anterior–posterior axis of *D. melanogaster* egg chambers; N is required at both poles for the correct expression of anterior–posterior markers [2,5,31]. The same has recently been described in *B. germanica* [32], suggesting the involvement of BgEGFR in the planar polarity deficiencies of FCs seen after BgN depletion.

Although BgDl and BgSer are the canonical ligands involved in the activation of the Notch pathway, other non-canonical ligands might participate in signalling [5,33]. In the ovary of *B. germanica*, the above canonical ligands showed complementary mRNA expression patterns, suggesting different ligand activity occurs at different times. In *D. melanogaster* oogenesis, Ser plays no role in the induction of N activity [10], the opposite to that seen in spermatogenesis, in which N is activated by Ser in the embryonic gonad [34]. In this work*,* the modulation of this ligand could not be confirmed, because no dramatic phenotypes were obtained after BgSer depletion, despite the drastic fall in the transcription of BgSer mRNA. However, BgSer is expressed in the ovary, and this expression is significantly altered when BgN levels are modified by dsBgN, dsBgHpo or dsBgEGFR treatment [16,32]. After dsBgSer treatment, the FCs were shorter, suggesting a functional action in the follicular epithelium of panoistic ovary. Thus, in the ovary of *B. germanica*, BgN appears to regulate the expression of BgSer, but the ligand does not affect the expression of the receptor.

The expression of Dl protein has been noted restricted to the germline cells in those insect species in which the Notch pathway has been studied [13–15,26]. In these species, Dl sends a signal from the oocyte which activates N in the FCs, thus controlling both the cell cycle and cell differentiation [19]. In this work, however, BgDl was detected in both germ and somatic cell lines, and was clearly absent in the posterior FCs. Further, after the activation of the Notch pathway resulting from BgHpo silencing, BgDl was overexpressed even in the posterior FCs, suggesting that somatic N–DI signalling determines the phenotype of the posterior FC population. It is known that N signalling induced by the ligands can be achieved by activating or inhibiting the receptor, depending on the developmental context [3,6].

The role of the Notch pathway in stalk formation is conserved from panoistic to telotrophic and through to polytrophic meroistic ovary types [15,16,19,21]. In the case of *D. melanogaster* and *T. castaneum*, the phenotypes resulting from N and Dl depletion are similar [2,13], though they are more severe for Dl depletion in *T. castaneum* [15]. However, neither the depletion of BgDl, nor of BgSer, nor even the simultaneous depletion of both, led to the absence of the stalk seen with BgN depletion. It is possible that small amounts of BgDl are enough to trigger a positive response of the pathway, as suggested by the different degrees to which the ovarioles are affected in dsBgDl-treated adult females. Thus, subtle changes in Dl levels may lead to differential responses in the follicles. This in turn suggests that non-canonical proteins are involved in the transmission of the signal, or that N acts independently of the ligand signal, as occurs in the *D. melanogaster* ovary when the Dl and Ser ligands are removed [35].

Notch signalling in the panoistic ovary of *B. germanica* allows for the proper development of the ovarian follicle by regulating both the proliferation and differentiation of somatic cells. The present results reveal the role of the Notch pathway in maintaining the proliferative and non-apoptotic state of FCs, as well in the differentiation of the posterior FC population. The role of N signalling cannot be fully understood' however, unless the spatio-temporal interplay between the Notch and other signalling pathways is taken into account, along with the dynamics of their canonical and non-canonical components. Work along these lines is presently in progress at our laboratory.

## 4. Material and methods

### 4.1. Cockroach colony and animal sampling

Freshly ecdysed sixth-instar nymphs and freshly ecdysed adult females of the cockroach *B. germanica* (L.) were obtained from a colony fed on Panlab dog chow and water ad libitum, kept in the dark at 29 ± 1°C and 60–70% relative humidity. The new adult females were maintained with males to ensure their having mated before their use in all experiments (the presence of spermatozoa in the spermathecae was assessed at the end of all experiments to confirm mating had occurred). Dissections and tissue sampling were performed on carbon dioxide-anaesthetized specimens, held under Ringer's saline. Nymphs and adults were examined at different ages (as shown in Results).

### 4.2. RNA extraction and expression studies

Total RNA was isolated using the GenElute mammalian total RNA kit (Sigma, Madrid, Spain). About 400 ng from each RNA extraction was DNAse treated (Promega, Madison, WI) and reverse transcribed with superscript II reverse transcriptase (Invitrogen, Carlsbad, CA) and random hexamers (Promega). RNA quantity and quality was estimated by spectrophotometric absorption at 260/280 nm in a NanoDrop ND-1000^®^ spectrophotometer (NanoDrop Technologies, Wilmington, DE).

Expression patterns for *B. germanica* BgN, BgDl and BgSer were determined by quantitative real-time PCR (qRT-PCR) in sixth-instar nymphs and adults in the first gonadotrophic cycle. Pools of two to six ovary pairs were used in each experiment. PCR primers for use in qRT-PCR expression studies were designed using Primer 3 v. 0.4.0 software [36]. The actin-5c gene of *B. germanica* was used as a reference for expression studies, and that of the eukaryotic initiation factor 4A (BgEIF4a) for functional studies. PCRs were performed as previously described [37]. Primer sequences and the accession numbers the sequences used are shown in the electronic supplementary material, table S1.

### 4.3. RNAi experiments

dsRNAs were synthesized as previously described [38]. dsBgN (363 bp) was designed to lie in a region that encompassed a fragment of the BgN Ankyrin domain [16]. dsBgDl (339 bp) was designed to lie in the N terminus of the N ligand, and dsBgSer (260 bp) was designed to lie in a region with no conserved domain. These dsRNAs were injected at a dose of 1 µg µl^−1^ into 6-day-old last (sixth) instar nymphs. To deplete both N ligands, a double injection of dsBgDl and dsBgSer was performed—dsBgDl first and dsBgSer 4 h later—on the opposite side of the abdomen. dsBgHpo, designed following a previous protocol [16], was injected (also into the side of the abdomen) into freshly ecdysed nymphs (day 0 of the sixth-instar). The electronic supplementary material, table S1 shows the primer sequences used to synthesize the dsRNAs.

### 4.4. Whole-mount immunolocalization

Ovaries were dissected from the last-instar nymphs and adults of different ages. Fixing and staining were performed as previously described [16,39]. The primary rabbit antibodies employed were anti-PH3 and anti-cleaved caspase-3 (Asp175) (Cell Signaling Technology, Denver, MA; dilution 1 : 250). The primary mouse antibodies used were anti-NICD (C17.9C6, Notch intracellular domain), anti-Delta (C594.9B, Delta extracellular domain) anti-Eya (eye absent; eya10H6, which appeared as good marker of the oocyte nucleus) and anti-β-tubulin (E7 tubulin beta), all obtained from the Developmental Studies Hybridoma Bank (Department of Biology, University of Iowa, Iowa City, IA; dilutions 1 : 100 for anti-NICD and anti-Delta, and 1 : 50 for anti-Eya and anti-β-tubulin, made from concentrated stocks). Tissues were washed with PBTBN three times and incubated for 2 h with either Alexa-Fluor 647 conjugated donkey anti-rabbit IgG or Alexa-Fluor 488 conjugated goat anti-mouse IgG secondary antibody (Molecular Probes, Carlsbad, CA), both diluted at 1 : 400 in PBTBN. These ovaries were then incubated for 20 min in 300 ng ml^−1^ phalloidin-TRITC (Sigma), and then for a further 5 min in 1 µg ml^−1^ DAPI (Sigma) in PBT. After three washes with PBT, the tissues were mounted in Mowiol (Calbiochem, Madison, WI), and observed using a Zeiss AxioImager.Z1 (Apotome) microscope (Carl Zeiss Microimaging).

### 4.5. Statistical analysis

Data are expressed as means ± s.e. Morphometric differences in ovarian follicles and follicular cells between treated and control individuals were analysed by using the Mann–Whitney test (performed using GraphPad Prism 6 Demo software, La Jolla, CA). Differences between expression levels were examined using the pair-wise fixed reallocation randomization test (performed using the Relative Expression Software Tool (REST) v. 2.0.7; Corbett Research, Sydney, Australia). This test makes no assumptions regarding data distribution [40].

## Supplementary Material

Irles Supplementary Material
