# Supplementary material for: The Notch pathway regulates both the proliferation and differentiation of follicular cells in the panoistic ovary of Blattella germanica
Source: Open Biol. 2016 Jan 13;6(1):150197. doi: 10.1098/rsob.150197 (PMC4736824; doi:10.1098/rsob.150197)
Supplement: Irles Supplementary Material [file rsob150197supp1.pdf]

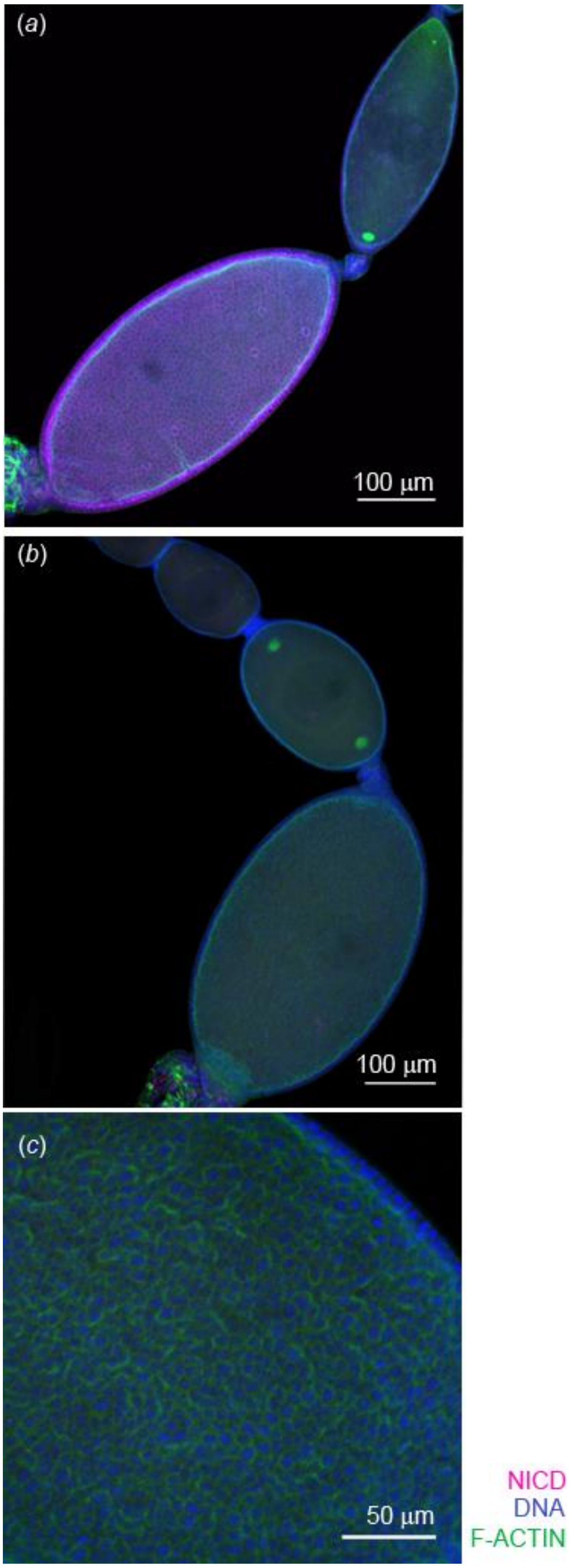

Figure S1. Mild phenotype in 0-day-old dsBgN-treated females.
(*a*) Ovariole from a dsMock-treated female showing high labelling for NICD in the basal ovarian follicle. (*b*) Ovariole from dsBgN-treated females showing a slightly phenotype after the depletion of BgN. The shape of the basal ovarian follicles remains similar to that of dsMock-treated females, but the NICD labelling was highly depleted. (*c*) Detail of the follicular epithelium in dsBgN-treated females showing the cytoskeleton arrangement in the follicular cells, the absence of mitosis and no labelling for NICD. DAPI (Blue) was used to DNA staining and TRITC-phalloidin (green) was used to stain F-actin microfilaments. In (*a)* and (*b)* the posterior pole of the basal follicle is towards to bottom.

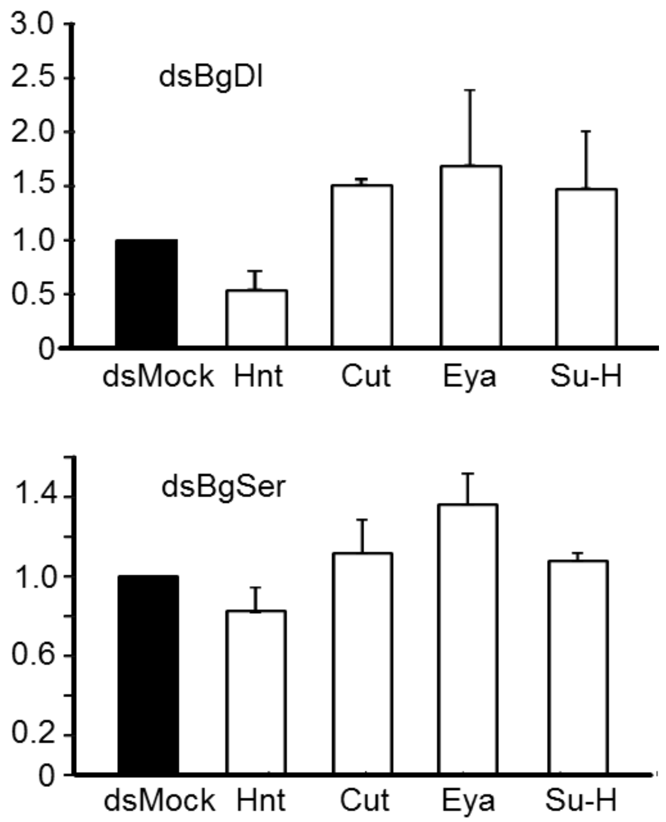

Figure S2. Relative expression of BgN, BgSu-H, BgDl and BgSer mRNAs in ovaries from dsMock, dsBgDl and dsBgSer-treated females. Females were treated in the last nymphal instar (6-day-old) and ovaries dissected in adult stage (5-day-old). Data represent normalized values against the control (reference value = 1) (n = 3).

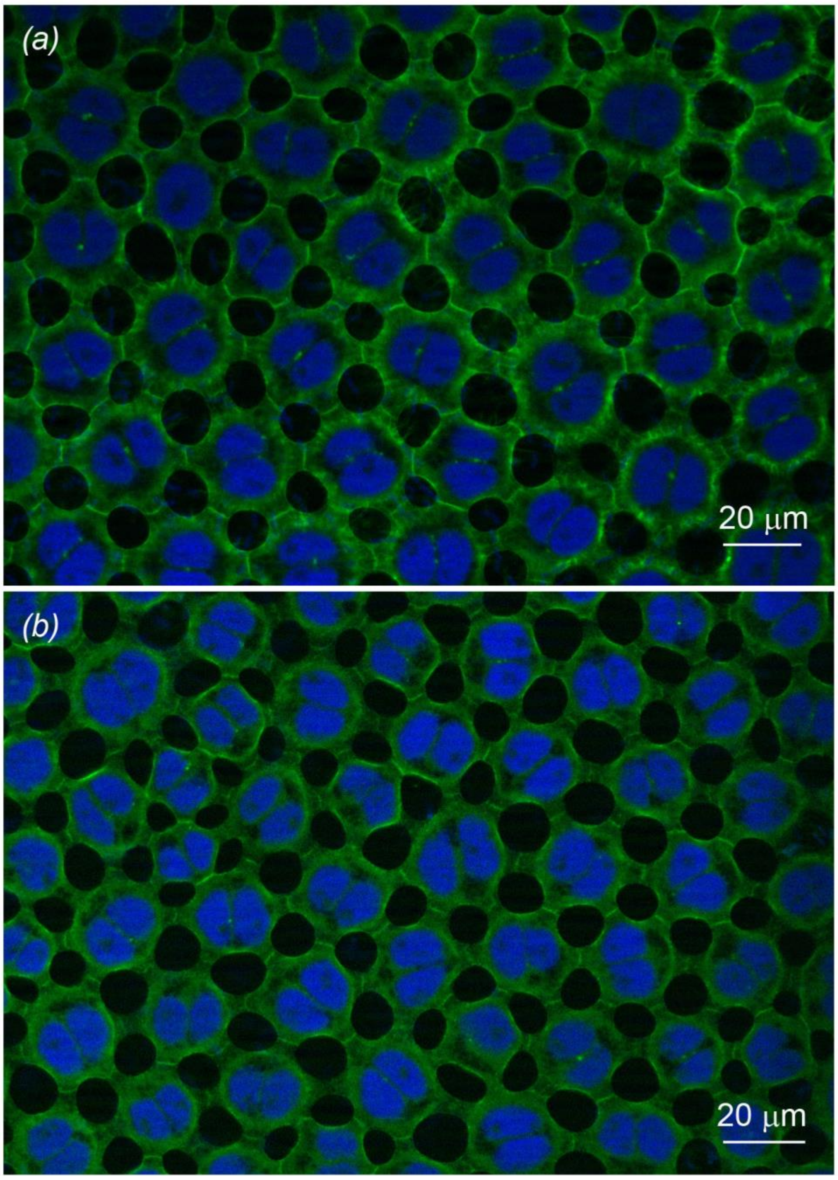

Figure S3. FCs from a basal ovarian follicle in 5-day-old adult females. (*a*) FCs in dsMock-treated females. (*b*) Smaller FCs in dsBgSer-treated females. DAPI (Blue) was used for DNA staining and TRITC-phalloidin (green) was used to stain F-actin microfilaments

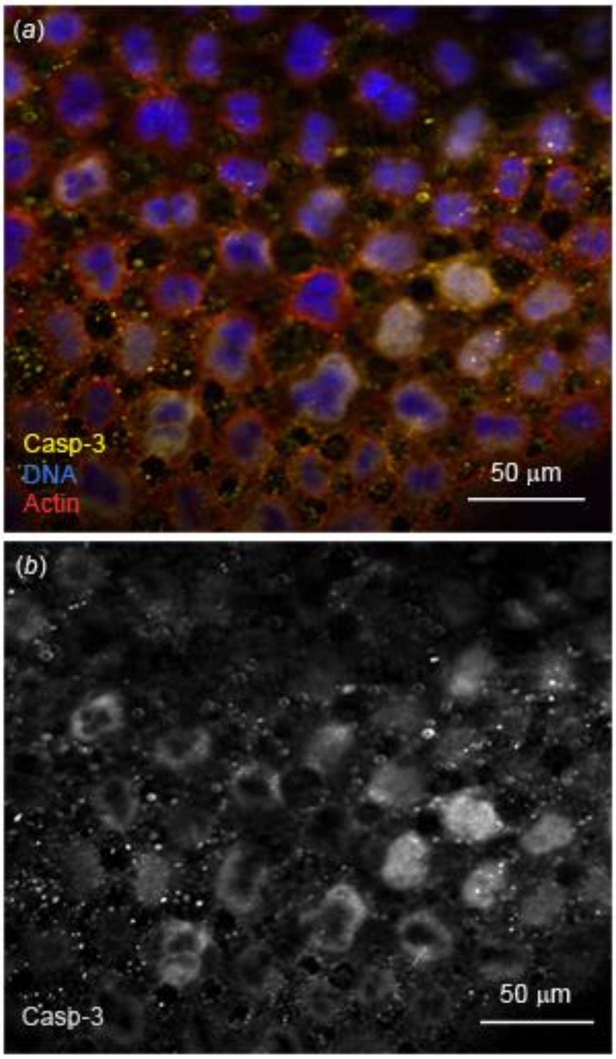

Figure S4. Follicular cell death in basal ovarian follicles from a 5-day-old adult female treated with dsBgSer. (*a*) DAPI (Blue) was used for DNA staining and TRITC-phalloidin (red) was used to stain F-actin microfilaments. An antibody anti-cleaved caspase-3 (casp-3, yellow) was used to label apoptotic FCs. (*b*) Chanel for anti-cleaved caspase-3 alone. FCs from dsMock-treated females were showed in figure 6*a and a''.*

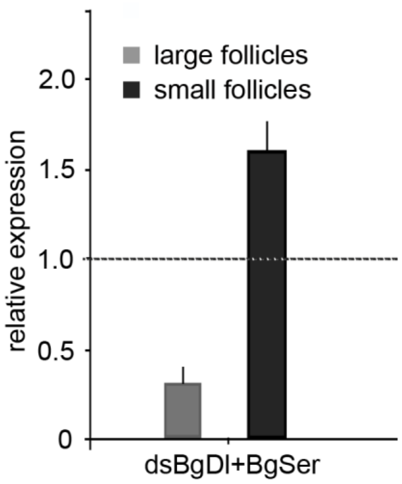

Figure S5. Expression of BgSer in ovaries from 5-day-old adult, double-ligand knockdown treated females. The mRNA expression for BgSer presented in figure 6*a,* here is split in accordance to the size of the ovarian follicles.

Table S1: Accession Number of Studied Sequences and Primer Sequence Used for qRT-PCR and RNAi experiments

	Accession number	Primer name		Primer sequence	Amplicon Length (bp)
1	HF969251	BgHpo-RT	F<br>R	5'-GACATTTGGAGCCTTGGCAT-3'<br>5'-AGGTTTCCCTTCAGCCATTTC-3'	51
2	HF969255	BgN-RT	F<br>R	5'-GCTAAGAGGCTGTTGGATGC-3'<br>5'-TGCCAGTGTTGTCCTGAGAG-3'	55
3	HF969255	BgN-RNAi	F<br>R	5'-CTCAGGACAACACTGGCAGA-3'<br>5'-AGGCTTCGTAACTGCCTTCA-3'	363
4	HF969256	BgDl-RT	F<br>R	5'-CCACTACAAGTGTTCGCCAA-3'<br>5'-TACCTCTCGCATTCGTCACA-3'	180
5	HF969256	BgDl-RNAi	F<br>R	5'-CAACGACTATGGCAAGGACA-3'<br>5'-ATCAGCGTCTCTCCTCCTGA-3'	339
6	HG515375.1	BgSer-RT	F<br>R	5'-TCCTCTTGGCAGTGCATTTG-3'<br>5'-CTTGATCACAGAGGATGCCG-3'	55
7	HG515376.1	BgSer-RNAi	F<br>R	5'-CCATGGGGTGAATGTCGTGA-3'<br>5'-CCTTGCTTCAGGTCACAGAG-3'	260
8	HF969267.1	BgCyc-E-RT	F<br>R	5'-TACCTGGGCAAATCCAAGAG-3'<br>5'-TTCATCTCGCTCATGACTGG-3'	76
9	LN812812	BgCasp1-RT	F<br>R	5'-AAGCGGAAGGATTCATACCA-3'<br>5'- GATGACTGCCTTGCCTCTTC-3'	80
10	HF969265	BgCut-RT	F<br>R	5’-AAATATGTGCTCGGCCTGTC-3’<br>5’-TGCATCTTGCGGTAACTGTC-3’	107
11	HF969258	BgHnt-RT	F<br>R	5’-CTACGACATCGCAAGAAGCA-3’<br>5’-AAAGGGCAGTGGAGTTGTTG-3’	40
12	HF969257	BgEya-RT	F<br>R	5’-GGCTCTTAGGCACAAAACGA-3’<br>5’-GCAAGGGCTGGAACTAACTG-3’	166
13	LN812813	BgSu-H-RT	F<br>R	5’-TGCCACACCAGTTTGGTTTA-3’<br>5’-TGGCCTGTACAACATTGAGC-3’	101
14	AJ862721	BgActin-5c	F<br>R	5'-AGCTTCCTGATGGTCAGGTGA-3'<br>5'-ACCATGTACCCTGGAATTGCCGACA-3'	213
15	HF969254	BgEIF4a	F<br>R	5'-ATGGTGACATGCCACAAAAA-3'<br>5'-GCAACACCTTTCCTTCCAAA-3'	208

F: Primer forward - R: Primer reverse
In red is showed the housekeeping genes: BgActin-5c used for pattern expression studies and EIF4-a used for mRNA expression in RNAi studies.
